# Recreation, transportation or labour saving? Examining the association between household asset ownership and body mass index among Ghanaian women

**DOI:** 10.1186/s40608-015-0075-z

**Published:** 2015-11-12

**Authors:** Fidelia A. A. Dake, Kamil Fuseini

**Affiliations:** Regional Institute for Population Studies, University of Ghana, P.O. Box LG 96, Legon, Accra Ghana; Population Training and Research Unit, North-West University (Mafikeng Campus), Mafikeng, South Africa

**Keywords:** Household asset ownership, Body mass index, Ghanaian women

## Abstract

**Background:**

In most of the developing world, ownership of modern household assets such as television sets, refrigerators, microwave ovens and washing machines is becoming common. Ownership of these household assets, however, promotes sedentary behaviour which has implications for obesity and non-communicable disease conditions such as hypertension, diabetes, stroke and other cardiovascular conditions. Additionally, increased household asset ownership is an indicator of socio-economic status and is also associated with obesity promoting dietary behaviours especially in urban areas. Very few studies have examined the relationship between household asset ownership and obesity in sub-Saharan Africa where asset ownership is becoming a norm. This paper examined the relationship between ownership of different types of household assets and Body Mass Index (BMI) among a nationally representative sample of Ghanaian women.

**Methods:**

The study analysed secondary data from the 2008 Ghana Demographic and Health Survey (GDHS) involving a total of 4916 women aged 15–49 years. The analytical sample consist of 4010 (weighted sample) non-pregnant females who had valid data on all the variables used in the analyses. The BMI of the women was used as the dependent variable with three categories of normal weight, overweight and obese based on the standard World Health Organization (WHO) classifications. Multinomial logistic regression models were specified to examine the relationship between ownership of household assets and BMI.

**Results:**

Obesity was more common among women whose household owned a television-15.60 %, DVD/VCD-18.58 %, computer-20.70 %, refrigerator-17.16 % and washing machine-27.43 %, but less common among women whose household owned a motorcycle/scooter-7.74 % and a bicycle-7.92 %. Household ownership of DVD was significantly associated with increased odds of obesity (OR = 1.59, *P* < 0.01) while ownership of a motor cycle/scooter (OR = 2.05, *P* <0.001), a refrigerator (OR = 1.33, *P* < 0.05), and a television set (OR = 1.27, *P* < 0.10) were associated with higher odds of overweight. Ownership of each additional household asset was also associated with 15 % and 25 % higher odds of overweight and obesity respectively.

**Conclusion:**

The findings indicate that at least one asset among the various domains of household assets examined is associated with either overweight or obesity among Ghanaian women. Also, increased household asset ownership is associated with increased odds of overweight and obesity. Interventions that aim at reducing sedentary and unhealthy dietary behaviours in the phase of increasing asset ownership maybe helpful in addressing the rising prevalence of obesity among Ghanaian women.

## Background

Over the last three decades, there has been a steady increase in the prevalence of obesity in all regions of the world including sub-Saharan Africa (SSA). Even though SSA is among the regions with the lowest prevalence of obesity, the rate of increase in the prevalence of obesity is among the highest in recent times [1]. Changing modes of transportation coupled with changes in work and recreational physical activity are reported to be among the major drivers of the current global obesity epidemic [2, 3]. Gradual change in daily routines from activities such as walking for transportation and engaging in physically tasking household activities, to the use of cars and automated domestic appliances have resulted in lower levels of physical activity, which may be contributing to the global rise in the prevalence of obesity [4–6].

Ownership of automobiles, modern household appliances and labour saving equipment such as washing machines, microwave ovens, refrigerators and food processors is gradually increasing in developing countries [4, 7]. This is as a result of improved socio-economic status and the rapid rate of urbanization in developing countries over the last three decades. For example, about 14 per cent of households in China acquired a motorized vehicle between 1989 and 1997 [2]. Furthermore, Du et al. [8] found a considerable increase in ownership of television sets in China within an eight year period (1989–1997), with close to 90 per cent of Chinese households owning a television set in 1997. This increase in television ownership in China was found to be greater in rural areas and among low income groups [8].

Increased asset ownership and reduced physical activity are some of the changes countries go through as part of socio-economic development. These changes accompany changes in dietary patterns in a process first described by Barry Popkin as the nutrition transition [9]. The nutrition transition is defined as the changes in dietary patterns and nutrient intakes when populations adopt modern lifestyles during economic and social development, urbanization and acculturation [10, 11]. The nutrition transition has been observed in dietary changes of rural and urban Africans in the Transition and Health during Urbanization of South-Africans (THUSA) study [12]. Additionally, the nutrition transition is underway in Tanzania where the obesity epidemic shows an indication of changing food consumption habits [13]. Several other studies show changes in dietary patterns characterising an ongoing nutrition transition in Africa [14–18].

Ownership of modern technological devices has been found to be associated with weight gain in several regions of the world [19] due to increasing physical inactivity. There are also indications of declining levels of physical activity in most developing countries [3]. Access to modern technological devices, including domestic appliances which impact physical activity have been suggested as contributing to rising obesity levels in the Global South, yet very few studies have empirically investigated the association between household asset ownership and obesity particularly in SSA. The present study examined this relationship in Ghana where household asset ownership and obesity are both on the rise.

Household asset ownership in Ghana has seen a marked increase over the last twenty years with ownership of most assets being relatively higher in urban compared to rural areas. Also, while ownership of some assets such as television sets, computers and smart phones have increased, others such as radio sets have seen a slight decline. The early rounds of the demographic and health surveys show close to a 50 per cent increase in the proportion of households owning a television set from 9 per cent in 1988 to 17 per cent in 1993 [20]. The Ghana Living Standard Survey (GLSS) round six report also shows that ownership of a television set almost doubled (from 40 to 75 %) in urban households and tripled (from 12 to 34 %) in rural households over a fifteen year period (from 1998/99–2012/13) [21]. Over the same period, ownership of cars increased slightly in both urban and rural areas but was predominantly higher in urban areas at 7 per cent in 2012/13 compared to 2 per cent in rural areas. On the other hand, ownership of bicycles continues to remain relatively higher in rural areas compared to urban areas [21].

Accompanying this rise in the ownership of household assets among the Ghanaian population is an increase in the prevalence of obesity and low levels of vigorous physical activity especially among women [22, 23]. Over a fifteen year period, the prevalence of obesity has increased from about 3 per cent in 1993 to about 10 per cent in 2008 among women aged 15 to 49 years [23]. Several studies have examined obesity among the Ghanaian population from a socio-demographic, lifestyle or medical perspective [24–26]. However, not many studies have investigated obesity from a socio-economic development perspective. This paper investigates the problem of obesity through the lenses of socio-economic development using household asset ownership as a proxy indicator.

## Methods

### Source of data

This study uses secondary data from the 2008 Ghana Demographic and Health Survey (GDHS). The GDHS is a nationally representative sample survey which is conducted every five years. The 2008 GDHS is the fifth round in the series and the most current publicly available data set. Permission to use the data was obtained from Measure DHS. Respondents for the 2008 GDHS were selected using a two-stage sampling design. At the first stage, a total of 412 clusters were selected nationwide using an updated master sampling frame constructed from the 2000 Population and Housing Census. The clusters were selected with probability proportional to size. At the second stage, thirty households were sampled from each of the selected clusters. Eligible individuals in the selected households were identified and interviewed. Informed consent was obtained from the respondents before being interviewed. The 2008 GDHS sampling procedure and methodology has been described in detail elsewhere [27].

### Study participants and sample size

The participants in this study were females aged 15–49 years who were usual residents of the selected households. A total of 5096 females were identified to be eligible out of which 4916 were successfully interviewed. Respondents who were pregnant, those who had missing or flagged BMI values and those who were underweight were excluded from the analyses. The analytic sample consist of 4010 (weighted sample) non-pregnant females who had valid information on all the variables used in the analyses.

### Variables

#### Dependent variable

The BMI of the respondents was used as the dependent variable. BMI is computed by dividing weight in kilograms by height in metres squared. The BMI method is used internationally as the standard of defining overweight and obesity. It is widely used for population level analysis of obesity in adult populations [28]. In this study, the standard World Health Organization (WHO) BMI cut-off points was used to categorise the study participants into three categories. Respondents with BMI values in the range of 18.50–24.99 kg/m^2^ were classified as being of normal weight, those who’s BMI were within the range of 25.00–29.99 kg/m^2^ were classified as being overweight and those with BMI ≥ 30.00 kg/m^2^ were classified as being obese. Women who were underweight (BMI <18.50 kg/m^2^) were excluded from the analyses.

#### Independent variables

Ownership of different types of household assets was used as the main independent variable and was measured based on whether or not a household had a television, computer, DVD/VCD, car/truck, motorcycle/scooter, bicycle, refrigerator, and washing machine. The assets were grouped into three main categories of; (1) recreational (2) transportation and (3) labour or energy saving. Recreational assets comprised of television set, computer and DVD/VCD. Transportation assets comprised of car/truck, motorcycle/scooter and bicycle. Refrigerator and washing machine were classified as labour/energy saving assets. The assets were also used differently in two separate analyses procedures. In the first stage of the analyses, the assets were used as separate independent variables while in the second stage, the individual assets were combined into a household asset index. The index ranged from 0 to 8 representing households owning none or all of the assets respectively.

#### Control variables

The study controlled for respondents’ level of physical activity and sedentary behaviour (measured using frequency of watching television). Socio-demographic characteristics of the respondents including age, highest level of education, current marital status, region of residence, type of place of residence and parity were also controlled for in the analyses.

### Methods of analysis

Exploratory data analyses was conducted on all the variables using frequencies/percentages and means for categorical and continuous variables respectively. Bivariate descriptions of the distribution of the independent variables across BMI categories were examined. Chi-square test was used to examine the significance of the bivariate associations. A multinomial logistic regression model was specified to assess the influence of household asset ownership on BMI controlling for other variables. The multinomial logistic approach was used because BMI was treated as a categorical dependent variable rather than an ordinal variable. Respondents were grouped into three categories of normal weight, overweight and obese based on their BMI. The three BMI categories were treated as separate categories thus making the use of the multinomial logistic regression model appropriate.

Two models were specified: the first model examined the influence of each household asset as a separate independent variable on BMI while the second model examined the effect of the aggregate household asset index (as a continuous variable) on BMI. The normal weight category was used as the reference category for the dependent variable in the regression models. The analyses were performed using Stata version 12.1. In order to ensure national representativeness and correct for non-response while also accounting for the complex survey design, the data was weighted using the ‘svyset” command.

## Results

About one-third (32.52 %) of the women were either overweight or obese. Specifically, about one in five were overweight while about one in ten were obese (Table 1). Among all the assets examined, television ownership was most common. Close to half of the women reported that their household owned a television set and a little over a quarter reported having a DVD/VCD in their household. Ownership of a computer was low, less than one-tenth of the women reported having a computer in their household. Among the assets classified under transportation, bicycle ownership was more common (29.69 %) compared to car/truck (8.70 %) and motorcycle (5.91 %). Only a few (0.75 %) of the women reported having a washing machine in their household. Examining the household asset index reveals about one-third (33.03 %) of the women belonging to households that do not own any of the assets under consideration (Table 1).

**Table 1:** BMI distribution of the study sample and types of assets owned by their household

	Percentage (%)
BMI category	
Normal weight	67.48
Overweight	22.40
Obese	10.12
Type of household asset (owned)	
Recreation	
Television	47.85
DVD/VCD	28.33
Computer	6.71
Transportation	
Car/Truck	8.70
Motorcycle	5.91
Bicycle	29.69
Labour saving	
Refrigerator	30.89
Washing machine	0.75
Household asset index	
Categorical	
0	33.03
1	23.80
2	15.53
3	14.24
4	7.86
5	3.74
6	1.46
7	0.33
Continuous (Mean)	1.59
Total N (weighted)	4010

Source of data: Ghana Demographic and Health Survey, 2008

The results of the bivariate analysis reveal a significant association between household asset ownership and BMI with motorcycle and bicycle both being significant at *P* <0.10 (Table 2). Obesity was three times more common among women who reported ownership of a television set in their household compared to their counterparts who did not (Table 2). Similarly, while about 7 % of the women from households that did not own a DVD/VCD were obese, close to 19 % of their counterparts from households that owned a DVD/VCD were obese. A similar pattern was observed for ownership of a computer. In the domain of transportation assets, obesity was more common among women in households that owned a car/truck and less common among women from households that owned a motorcycle and a bicycle. The proportion of overweight and obese women was highest among women from households that owned a refrigerator and a washing machine at approximately 30 and 27 per cent respectively (Table 2).

**Table 2:** Distribution of study sample by type of household asset and BMI

Type of household asset	Percentage (%) in BMI category			Chi-Square *P*-value
	Normal weight	Overweight	Obese	
Recreation				
Television				0.000
Yes	56.47	27.93	15.60	
No	77.59	17.32	5.09	
DVD/VCD				0.000
Yes	53.52	27.89	18.58	
No	73.00	20.23	6.78	
Computer				0.000
Yes	50.24	29.06	20.70	
No	68.72	21.92	9.36	
Transportation				
Car/Truck				0.000
Yes	58.68	23.06	18.26	
No	68.32	22.33	9.35	
Motorcycle				0.057
Yes	63.33	28.92	7.74	
No	67.74	21.99	10.27	
Bicycle				0.057
Yes	72.53	19.55	7.92	
No	65.35	23.60	11.05	
Labour saving				
Refrigerator				0.000
Yes	52.68	30.16	17.16	
No	74.09	18.93	6.98	
Washing machine				0.014
Yes	51.52	21.05	27.43	
No	67.60	22.41	9.99	

Source of data: Ghana Demographic and Health Survey, 2008

Controlling for frequency of watching television, physical activity and socio-demographic characteristics, the results show that television ownership increases the chances of being overweight by 27 per cent, significant at *P* < 0.10 (Table 3, Model 1). Ownership of a computer was also associated with a 35 per cent higher chance of overweight (*P* < 0.10). A households’ ownership of DVD/VCD was found to be associated with a 59 per cent higher likelihood of obesity (*P* < 0.01). The result with regards to car/truck ownership was opposite of what was expected. Women from households that owned a car/truck were significantly less likely (OR = 0.64) to be overweight compared to those whose household did not own a car. On the other hand, women from households that owned a motorcycle were two times as likely to be overweight compared to their counterparts from households that did not own a motorcycle. Increased household asset ownership was associated with increased odds of overweight and obesity (*P* < 0.001) with ownership of any one additional household asset being associated with a 15 % and a 25 % higher likelihood of being overweight and obese respectively (Table 3, Model 2).

**Table 3:** Results of multinomial logistic regression analysis examining the relationship between ownership of different household assets and BMI

	Odds ratio			
Type of household asset	Model 1		Model 2	
Recreation	Overweight	Obese	Overweight	Obese
Television ^[No]^	1.27^+^	1.39		
Computer ^[No]^	1.35^+^	1.51		
DVD/VCD ^[No]^	0.98	1.59**		
Transportation				
Car/truck ^[No]^	0.64**	0.80		
Motorcycle/scooter ^[No]^	2.05***	1.23		
Bicycle ^[No]^	1.10	0.98		
Labour saving				
Refrigerator ^[No]^	1.33*	1.19		
Washing machine ^[No]^	1.01	1.75		
Household asset index			1.15***	1.25***
Control variables				
Frequency of watching television ^[Not at all]^				
Less than once a week	1.19	1.65^+^	1.24	1.78*
At least once a week	1.12	1.51^+^	1.15	1.60*
Almost every day	1.29	2.07***	1.35*	2.33***
Number of physical activity days/week ^[0]^				
1–2	1.10	0.84	1.10	0.84
3–4	0.92	0.77	0.92	0.77
5–7	1.04	0.75	1.04	0.74
Age ^[15–19]^				
20–24	1.69**	1.47	1.71**	1.47
25–29	3.66***	4.42***	3.73***	4.47***
30–34	4.51***	8.63***	4.60***	8.64***
35–39	4.21***	14.91***	4.39***	14.68***
40–44	5.61***	22.89***	5.69***	22.09***
45–49	4.52***	17.50***	4.50***	17.08***
Education ^[No education]^				
Primary	1.43*	1.79*	1.45*	1.78*
Middle/Junior High School	1.38*	1.63*	1.39*	1.65*
Secondary/Senior High School	1.46^+^	1.59^+^	1.46^+^	1.60^+^
Higher	1.73*	1.62	1.72*	1.72
Current marital status ^[Never married]^				
In union	1.03	1.77*	1.02	1.82**
Formerly in union	1.25	2.20**	1.23	2.28**
Region ^[Greater Accra]^				
Western	1.18	0.68^+^	1.23	0.67^+^
Central	1.38	0.67	1.44+	0.68
Volta	1.14	1.00	1.19	0.93
Eastern	1.30	0.93	1.32	0.90
Ashanti	0.99	0.59**	1.04	0.59**
Brong Ahafo	0.63**	0.33***	0.68*	0.30
Northern	0.45***	0.23***	0.54**	0.20***
Upper east	0.53*	0.41*	0.59*	0.36*
Upper west	0.42***	0.19***	0.50***	0.17***
Place of residence ^[Rural]^				
Urban	1.85***	2.09***	1.89***	2.20***
Parity	1.04	0.99	1.04	0.99

Source of data: Ghana Demographic and Health Survey, 2008

[ ] Reference Category

^+^ *P* < 0.10,*P < 0.05, **P < 0.01, ***P < 0.001

Among the control variables included in the analyses, frequency of watching television exhibited a significant association with the likelihood of obesity. In both models, women who reported watching television almost every day had about a two times higher chance of being obese (*P* < 0.001) compared to their counterparts who did not watch television (Table 3). In Model 2, women who reported watching television less than once a week (OR = 1.78) or at least once a week (OR = 1.60) were significantly (*P* < 0.05) more likely to be obese. The women’s level of physical activity did not show a statistically significant association with their chances of being overweight or obese. Urban dwellers were about two times more likely to be overweight or obese (*P* < 0.001) compared to rural dwellers. And compared to the youngest age group (15–19), older age groups were significantly more likely to be overweight or obese except for the 20–24 age group which did not show statistical significance in the obese category (Table 3).

## Discussion

The findings of this study showed television ownership being most common. Obesity was also found to be more common among women whose household owned a television. In another study conducted in Ghana among a representative sample of women aged 18 years and older in Accra, Duda et al. [29] found that obese women were significantly more likely to own a television (and other assets including, a refrigerator, a telephone or a car) than their non-obese counterparts. Similar to the findings of the current study, Lear et al. [4] found television ownership to be more common in a study involving high, middle and low-income countries. Nurdiati et al. [30] also found obesity to be more common among women who owned a television set in Indonesia. In the present study, ownership of television was most common but was not significantly associated with increased odds of obesity as has been found in other studies involving high, middle and low-income countries [4]. Television ownership was, however, found to be associated with increased odds of overweight at *P* < 0.10 in the current study. It is worth mentioning that this association did not reach the *P* < 0.05 statistical significance level.

Television ownership has an inherent tendency of encouraging sedentary behaviour through increased watching/screen time. In this study, women who reported watching television almost every day were at increased risk of being obese compared to those who did not watch television. Among women in Ethiopia, those who reported watching television were significantly more likely to be overweight compared to their counterparts who did not watch television [31]. Similar results were also found among adult out-patients in Uyo in the Niger Delta region of Nigeria [32].

Similar to the findings of this current study, Lear et al. [4] found that ownership of additional household assets was associated with increased BMI in high, middle and low-income countries. Additionally, Lear et al. [4] also found that in low-income countries, there was a marked and stepped increase in the odds of obesity with increased asset ownership. Similarly, Banks et al. [19] found that among Thai adults, the risk of being obese increased significantly with increasing household ownership of appliances such as refrigerator, microwave ovens and washing machine even after adjusting for frequency of doing housework/gardening, smoking, consumption of alcohol, fried foods, Western type junk food, fruits and vegetables.

Asset ownership has been found to be associated with obesity implicating behaviours explaining why increased household asset ownership is associated with increased risk of overweight and obesity. Asset ownership affects physical activity in all forms including domestic, leisure-time and work-related physical activity [33]. With the increase in ownership of devices such as washing machines, household chores are becoming more mechanized rather than physically tasking [7, 34]. There is also a gradual shift from outdoor leisure-time physical activities to sedentary types of activity such as watching television [34]. With increased ownership of mechanized means of transportation, walking for travel is on a decline [35]. For example, among urban black Soweto women, Gradidge et al. [36] found that women who reported owning a motor vehicle walked less for travel and participated in more leisure-time activity while women who owned a television reported significantly lower moderate-vigorous physical activity and walking for travel. In a multi-country study involving sites in the United States (US), South Africa, Jamaica, Seychelles and Ghana, Shoham et al. [37] found that car owners show similar low levels of moderate-vigorous physical activity regardless of whether they lived in the US or in the other countries. Their study also found that participants in Ghana (and in Jamaica, Seychelles and US) whose household own a car had lower levels of physical activity than participants in the US whose household did not own a car [37].

There is evidence of reduced physical activity globally due to changing physical activity patterns. In the developing world, particularly in China and Brazil, Ng and Popkin [38] report high declines in physical activity and a shift towards sedentary activity in domains such as domestic physical activity which has been aided by greater access to home technologies including appliances. In a study conducted in Thailand, Banks et al. [19] found that the risk of being obese was significantly higher in men and women from households that owned energy saving appliances including a refrigerator, microwave oven and washing machine.

Increased household asset ownership is an indicator of improved socio-economic status and is also associated with changes in dietary habits [39]. This is especially so in urban areas where Western type foods replace traditional staple foods [40–43]. The association between household asset ownership and BMI gives an indication of changes in dietary behaviours and physical activity associated with socio-economic development.

This study is among the few studies that examines the relationship between ownership of household assets and obesity in Ghana in the phase of improving socio-economic conditions. The findings provide supporting evidence of the relationship between household asset ownership and overweight/obesity in sub-Saharan Africa as has been reported in studies conducted in other regions [4, 19]. The study demonstrates that increased ownership of household assets including recreational, transportation and energy saving assets is associated with overweight and obesity even after adjusting for demographic and socio-economic factors and lifestyle behaviours including physical activity and sedentary behaviour.

The results need to be interpreted with caution taking into account some limitations. Firstly, the study cannot establish causality because of the use of cross-sectional data. Secondly, the study does not control for the dietary behaviour due to lack of data. These limitations notwithstanding, the findings of this study have important implications. In most developing countries, household asset ownership is an indicator of a household’s socio-economic status and has become a common way of assessing wealth. However, changes in physical activity and dietary behaviour brought on by socio-economic development may be contributing to the prevalence of obesity in developing countries. Tackling obesity in developing countries thus requires addressing behaviours such as physical inactivity and unhealthy dietary habits which accompany socio-economic development.

## Conclusion

The results of this study show that increased household asset ownership is associated with overweight and obesity among Ghanaian women. The results may be applicable to other developing countries, particularly those in sub-Saharan Africa where household asset ownership is increasing. Public health interventions aimed at reducing obesity should target reducing sedentary and unhealthy dietary behaviour in the phase of increasing household asset ownership.
